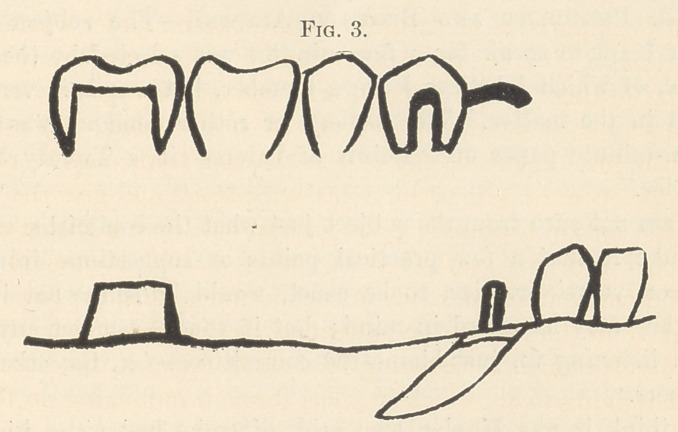# The Removable Bridge as a Conservator of Teeth

**Published:** 1903-09

**Authors:** A. C. Eglin

**Affiliations:** Philadelphia


### THE REMOVABLE BRIDGE AS A CONSERVATOR OF TEETH.[1]

BY DR. A. C. EGLIN, PHILADELPHIA.

In the broad field of bridge-work it is my intention to speak of that specific quality in removable work, which seems to have received the least attention from writers on the subject, but which is in reality one of its chief advantages,—namely, conservation.

In all cases where bridge-work is indicated the removable form can be used, and the facility with which it can be cleansed or repaired is generally recognized. There, as a rule, the credit given the class of work ends, but it should be borne in mind how thoroughly, when the bridge is removed, adjacent teeth can be cleansed, or, in case of necessity, the ease with which the surface of the tooth approximating the piece can be filled. Any one who has inserted a gold filling in the proximating side of the tooth next to a fixed bridge can speak feelingly of the difficulties attending such an operation.

The possibilities of the tube and split pin attachment as a con-

---

[1] Read before the Academy of Stomatology, Philadelphia, February 24, 1903.

servator of the natural crown are well worth your careful consideration.

Not infrequently we are called upon to supply the loss of a single anterior tooth. The particular case which I shall describe is the replacing of the first superior bicuspid. A most artistic dummy can be adjusted by opening into the cuspid root from the basilar ridge and enlarging the canal to receive a platinum tube. Into this tube is carefully fitted a split pin, bent in such a manner that it can readily be soldered to the bicuspid dummy. As a further support, a spur is soldered to the piece which rests in a gold filling in the sulcus of the second bicuspid. At this point my meaning may be made clearer by a rough sketch of the piece. (Fig. 1.)

Fig. 1.

The amputation of natural crowns of teeth, under whatever circumstances, gives the operator twinges of conscience, to say nothing of the feelings of the patient, who cannot be expected to appreciate why good sound teeth should be so mutilated. By using the method just described, any of the anterior teeth may be restored, and the operator will preserve his self-respect and give the patient a better crown, because it is the natural one, than a Richmond constructed with the greatest skill. Were a fixed Richmond crown, with dummy attached, made in such a case as this, the piece would, apart from the loss to the patient of the natural crown, still have the disadvantage of harboring food-stuffs between the dummy and the adjoining tooth, which in time, through impinging on the gum at the cervical margin, might cause an irritation of the cementum that in many cases could be treated only by the removal of the bridge.

A little bridge of three teeth made two years ago, in which only one root was crowned, demonstrates still further, by using another variation of the removable type, the conservation made possible by this method.

The case in point was to supply the loss of the first bicuspid and first molar on the left side of the inferior maxilla. The second bicuspid was crowned with double or telescoping caps, the outer cap having the first bicuspid and molar dummies soldered to it. As in the former case, spurs rested in gold fillings in the cuspid and second molar. With a fixed piece, to be practical, it would have been necessary to crown at least two teeth. (Fig. 2.)

Fig. 2.

The last case to be described presented the conditions seen in the drawing. The space to be bridged was between the second lower right molar and the first bicuspid root, the crown of which had

Fig. 3.

been lost for some time. The peculiar difficulty lay in the bicuspid root, which inclined towards the second molar at an angle of almost forty-five degrees from the perpendicular. To make use of this root, the ordinary attachment of a tube and split pin could not have been used, because the pin has always to be parallel, or nearly so, to the other abutment. To get over this trouble, it was necessary to reverse the usual order and solder the tube in the bridge and attach the split pin to the floor of the cap covering the root. To avoid undue strain upon the root, which owing to its position was none too strong, a heavy bar of iridio-platinum wire was soldered to the bridge and allowed to rest on the cuspid. (Fig. 3.)

Fixed work in this case would have meant the loss of the natural crown of the cuspid and also the extraction of the bicuspid root.

In a small way the fact has been impressed upon me that there are numerous cases where teeth may be saved by using the removable form of bridge. The natural crown is better than any artificial substitute which we can make, so let us not ruthlessly cut off crowns that may be made to serve the requirements of the case without amputation.

## Figures and Tables

**Fig. 1. f1:**
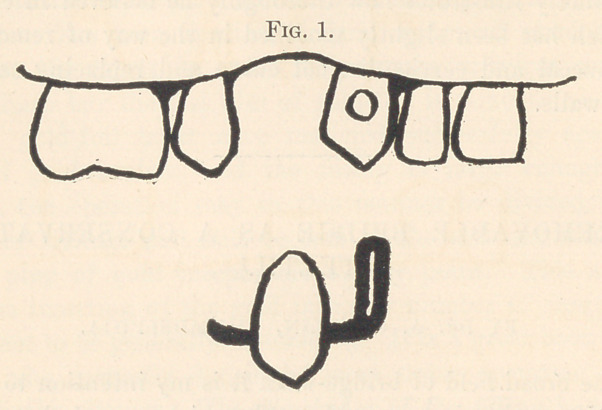


**Fig. 2. f2:**
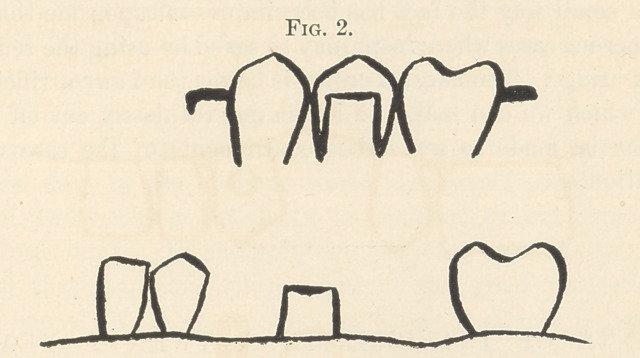


**Fig. 3. f3:**